# G9a Correlates with VLA-4 Integrin and Influences the Migration of Childhood Acute Lymphoblastic Leukemia Cells

**DOI:** 10.3390/cancers10090325

**Published:** 2018-09-12

**Authors:** Elena Madrazo, David Ruano, Lorea Abad, Estefanía Alonso-Gómez, Carmen Sánchez-Valdepeñas, África González-Murillo, Manuel Ramírez, Javier Redondo-Muñoz

**Affiliations:** 1 Department of Immunology, Hospital 12 de Octubre Health Research Institute (imas12), School of Medicine, Complutense University, 28040 Madrid, Spain; emadrazo@ucm.es (E.M.); e.alonso.gmz@gmail.com (E.A.-G.); 2 Department of Pediatric Hematology & Oncology, Hospital Universitario Niño Jesús, 28009 Madrid, Spain; druano64@hotmail.com (D.R.); lorea.abad@salud.madrid.org (L.A.); carmen.sanchez.val@gmail.com (C.S.-V.); manuel.ramirez@salud.madrid.org (M.R.); 3 Oncolohematology Unit, Hospital Universitario Niño Jesús, 28009 Madrid, Spain; africa.gonzalez@salud.madrid.org; 4 Health Research Institute La Princesa, 28006 Madrid, Spain; 5 Lydia Becker Institute of Immunology and Inflammation, Manchester Collaborative Centre for Inflammation Research, University of Manchester, Manchester M13 9PL, UK

**Keywords:** VLA-4, G9a, acute lymphoblastic leukemia, epigenetics, migration

## Abstract

Acute lymphoblastic leukemia (ALL) is the most common pediatric cancer. As ALL progresses, leukemic cells cross the endothelial barrier and infiltrate other tissues. Epigenetic enzymes represent novel therapeutic targets in hematological malignancies, and might contribute to cells’ capacity to migrate across physical barriers. Although many molecules drive this process, the role of the nucleus and its components remain unclear. We report here, for the first time, that the expression of G9a (a histone methyltransferase related with gene silencing) correlates with the expression of the integrin subunit α4 in children with ALL. We have demonstrated that G9a depletion or its inhibition with BIX01294 abrogated the ability of ALL cells to migrate through an endothelial monolayer. Moreover, G9a-depleted and BIX01294-treated cells presented bigger nuclei and more adherent phenotype than control cells on endothelial monolayers. Blocking G9a did not affect the cell cytoskeleton or integrin expression of ALL cell lines, and only its depletion reduced slightly F-actin polymerization. Similarly to the transendothelial migration, G9a inhibition impaired the cell migration induced by the integrin VLA-4 (α4β1) of primary cells and ALL cell lines through narrow spaces in vitro. Our results suggest a cellular connection between G9a and VLA-4, which underlies novel functions of G9a during ALL cell migration.

## 1. Introduction

Acute lymphoblastic leukemia (ALL), the most common cancer in children, is characterized by the accumulation of hematopoietic B- or T-cell precursors, which eventually infiltrate the bone marrow or thymus and secondary organs, resulting in leukemia progression [1,2]. ALL patients are stratified according to multiple subtypes defined by genetic abnormalities, such as chromosomal translocations, chromosomal deletions, amplifications, fusions, etc. [3]. Risk-tailored therapy protocols can cure most children with ALL but non-responding and relapsed pediatric ALL patients (20% of total) have poor prognosis and are the leading cause of cancer mortality at that age [4]. Most relapses occur in patients with no identifiable genetic alterations, i.e., the intermediate risk group [5]. ALL cells present specific genetic and epigenetic changes, which open new avenues to stratify patients and develop more effective therapies [6,7]. It has been reported that the methylation of specific histones is linked to cancer cell invasion [8,9]. One of these enzymes, the histone methyltransferase G9a catalyzes H3K9 methylation (a heterochromatin marker) [10], and is critical in lymphocyte development and leukemogenesis [11]; and its inhibition promotes apoptosis in acute leukemias [12]. The integrin α4β1 (CD49d/CD29, very late antigen-4, VLA-4) is a cell receptor that binds fibronectin, VCAM-1, osteopontin and other protein such as MMPs [13,14,15]. The expression of VLA-4 in pediatric ALL patients is associated with poor outcome and relapse-free survival (RFS) probabilities [16]. VLA-4 adhesion promotes G9a activity and H3K9 methylation during Jurkat (a T-ALL cell line) and normal lymphocyte cell migration [17]; however, the interplay between VLA-4 and G9a, and how they contribute to ALL dissemination, has not been described before.

The function and molecular connections of G9a during ALL cell migration are not known. These are important issues since G9a might represent a therapeutic target in ALL. We described a correlation between the gene expression of G9a and the α4 subunit of VLA-4 in samples from patients with ALL but not in healthy donors. Furthermore, G9a activity is critical during ALL transendothelial migration and ALL migration in response to VLA-4 adhesion. Together, our findings describe, for the first time, that G9a might play a direct role on ALL cell migration.

## 2. Results

### 2.1. Patient Characteristics

We studied samples from 50 children patients (age 1–14 years old) with a diagnosis of ALL, including patients carrying any genomic translocation and other bioclinical parameters. Major karyotypic and clinical characteristics, including sex, risk stratification, WBC (white blood cell) count, blasts in BM (bone marrow) and MRD (minimal residual disease) after induction are summarized in Table 1. 

### 2.2. The Expression of G9a Correlates with α4 Integrin Expression in ALL Cells

**Figure 1:**
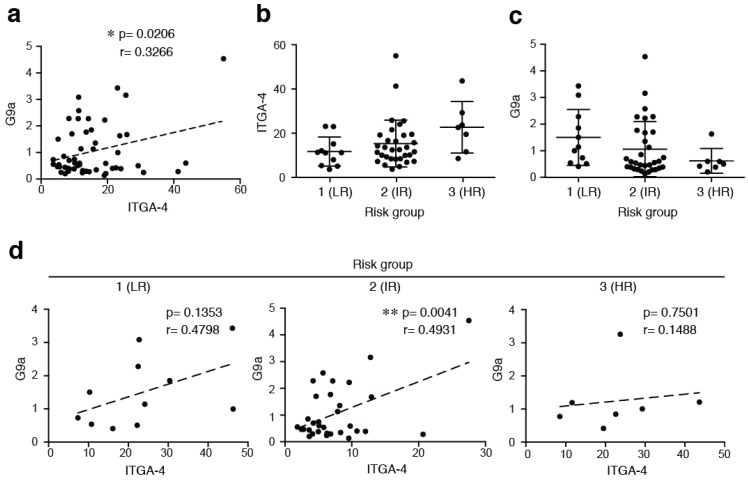
Expression and correlation of ITGA-4 and G9a in children patients of ALL. (**a**) ITGA-4 and G9a expression analyzed by RT-qPCR. Expression levels were normalized by TBP and graph shows the mean of children ALL patients (*n* = 50). Pearson’s correlation coefficient (*r*) and *p*-value between ITGA-4 and G9a are shown. * *p* < 0.05; (**b**,**c**) Patients were divided according to their risk groups (LR, low risk; IR, intermediate risk; HR, high risk) and ITGA-4 (**b**) and G9a (**c**) expression analyzed; (**d**) Patients were divided as in (**b**) and Pearson’s correlation coefficient (*r*) and *p*-value between VLA-4 and G9a are shown. ** *p* < 0.01.

The histone H3 is methylated by several enzymes from suv39h family, including Suv39h1 and G9a [18]. To elucidate the expression pattern of both histone H3K9 methyltransferases in childhood ALL, samples from pediatric patients with primary ALL and 10 samples from healthy donors were determined by using RT-qPCR. ITGA-4 (the integrin subunit α4), G9a and Suv39h1 expression was detected in all of the samples analyzed. We observed a significant Pearson correlation between ITGA-4 and G9a levels (*p* = 0.0206) but not with Suv39h1 (*p* = 0.1524) (Figure 1a and Figure S1a). Moreover, we did not find any correlation between G9a and ITGA-4 in a small cohort of healthy donors (Figure S1b). To further analyze the expression level of G9a according to the clinical risk grade groups, all patients were divided into three subgroups (1-low; 2-intermediate; and 3-high risk). We confirmed a tendency for high ITGA-4 expression levels to associate with high-risk group (Figure 1b). Interestingly, we found that G9a expression exhibited an opposite trend to ITGA-4 with clinical risk grade in ALL cells (Figure 1c). By determining the correlation between ITGA-4 and G9a levels within the different risk groups, we observed that intermediate-risk group presented a significant correlation between G9a and VLA-4 expression (Figure 1d). We stratified the patients according to their G9a expression into lower (LE) or higher (HE) than the median (Median = 0.6001) groups, confirming that the low-risk group showed more patients with HE of G9a whilst the high-risk group presented the opposite tendency (Table 2). Our results suggest that G9a and ITGA-4 levels present an opposite trend according to the different risk groups and may act jointly in children with an intermediate stage of ALL.

### 2.3. G9a Depletion Abrogates ALL Transendothelial Migration

**Figure 2:**
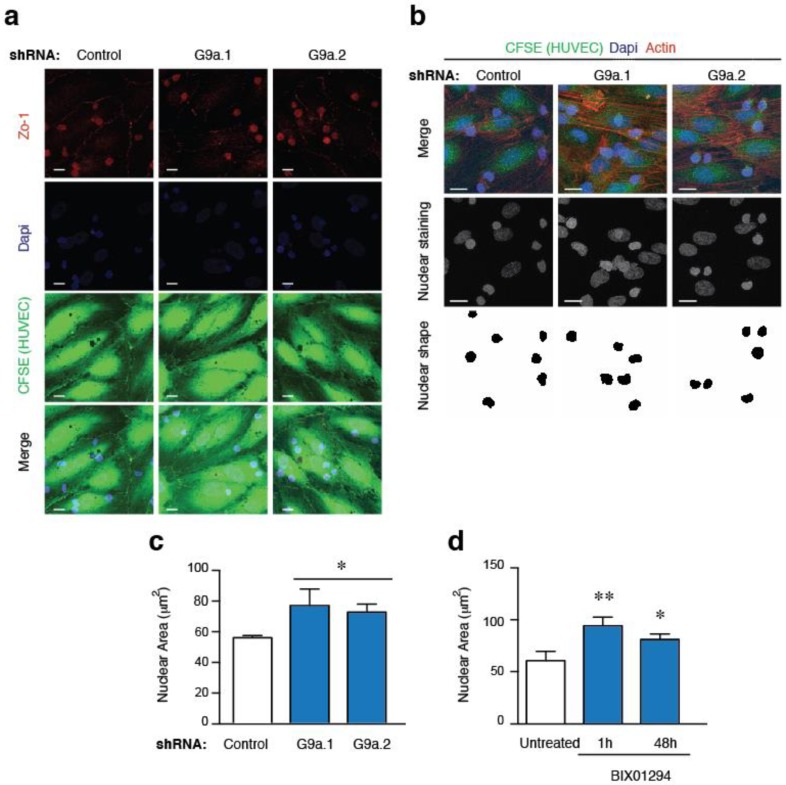
G9a depletion increases the nuclear area of ALL cells. (**a**) HUVEC cells were grown to confluency, labelled with CFSE and stimulated with TNFα for 16 h. Control or G9a depleted Jurkat cells were plated on TNFα-activated HUVEC cells. Cells were fixed, permeabilized and analyzed to visualized their nuclei (DAPI, blue), F-actin (Phalloidin, cyan), and endothelial junctions (Zo-1, red); (**b**) Control and G9a depleted Jurkat cells were cultured on CFSE labelled HUVEC activated with TNFα, fixed and stained for DAPI (blue) and F-actin (red). Nuclear shapes were determined; (**c**) Graph shows the nuclear areas quantified from (**b**); Mean *n* = 3 replicates ± SD. Bar = 10 μm. * *p* < 0.05; (**d**) Graph shows the nuclear areas from untreated or BIX10924 treated Jurkat at cells cultured on TNFα-activated HUVEC. Mean *n* = 3 replicates ± SD. * *p* < 0.05; ** *p* < 0.01.

Dissemination of ALL cells requires the extravasation of leukemia cells from the blood vessels across the endothelial barrier in a process known as transendothelial migration (TEM) [19]. To analyze the role of G9a in a more physiological context, we determined the cell capacity of two G9a depleted cell lines to cross a monolayer of human umbilical vein endothelial cells (HUVEC). HUVEC cells were stained with CFSE (which do not interfere with the TEM process) and the integrity of the monolayer was confirmed by staining for Zo-1, an endothelial cell-cell junctions marker. Firstly, we verified that control or G9a depleted cells were able to attach onto HUVEC cell monolayer (Figure 2a). We used the specific G9a inhibitor BIX01294, to block G9a activity for 1 h and 48 h, with no effects on cell attachment onto HUVEC cells (Figure S2a). By analyzing the nuclear shape of ALL cells onto HUVEC cells, we determined that G9a depletion or inhibition promoted a significant increment of the nuclear area of ALL cells (Figure 2b–d and Figure S2b).

**Figure 3:**
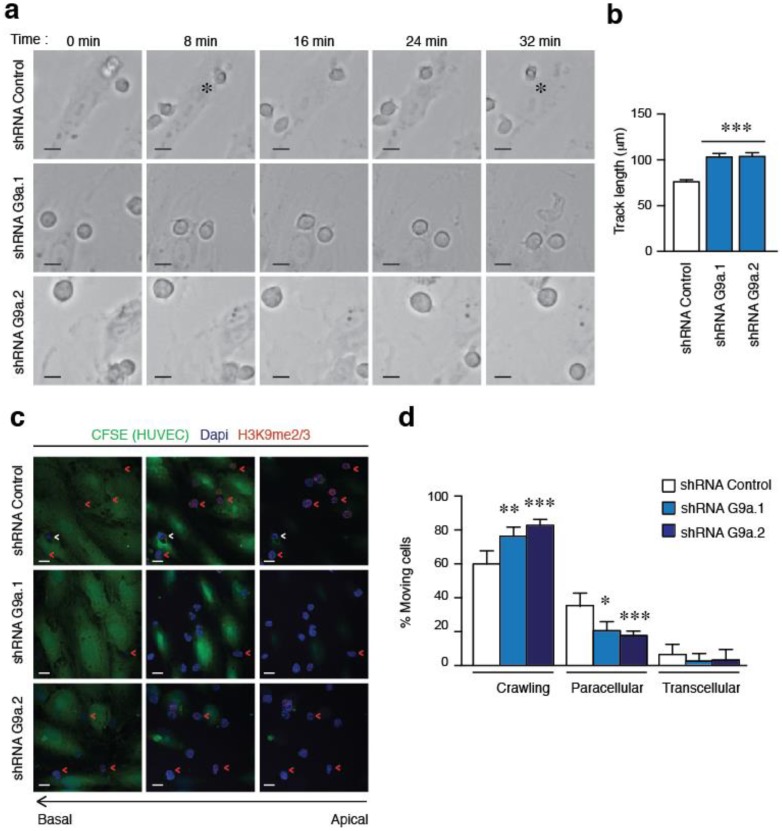
G9a depletion abrogates the TEM of ALL cells. (**a**) Representative images of control or G9a depleted Jurkat cells migrating on TNFα-activated HUVEC cells. Cells were tracked through time. Asterisk indicates a transmigrating cell. Bar = 10 μm (**b**) Control or G9a depleted Jurkat cells were labelled with CFSE to track their movement on TNFα-activated HUVEC monolayer. Graph shows the quantification of track lengths. Mean *n* = 200 cells ± SEM. *** *p* < 0.001; (**c**) Control or G9a depleted Jurkat cells were plated on TNFα-activated HUVEC cells labelled with CFSE. After 30 min, cells were fixed, permeabilized and analyzed to visualize their nuclei (blue), F-actin (cyan), and H3K9me2/3 (red). White arrows indicate cells undergoing transcellular TEM. Red arrows indicate cells crossing through cell-cell junctions in paracellular TEM; (**d**) Graph shows the percentage of control or G9a depleted cells crawling or performing TEM. Mean *n* = 5 ± SD. Bar = 10 μm. * *p* < 0.05; ** *p* < 0.01; *** *p* < 0.001.

We next investigated the contribution of G9a expression to ALL migration across HUVEC cells. Firstly, we confirmed by time-lapse that control cells were able to pass through the endothelial barrier (Video 1–3 in supplementary material) whilst G9a depleted cells remained crawling and extending multiple protrusions (Video 4 and 5 in supplementary material and Figure 3a). Interestingly, tracking of G9a depleted cells showed that they moved by crawling on endothelial monolayer more than control cells (Figure 3b). We confirmed that control cells showed higher levels of H3K9me2/3 staining compared to G9a depleted cells attached to HUVEC (Figure 3c). Then, we defined the position and migration of control or G9a depleted cells relative to the endothelial cell monolayer and quantified the number of cells crawling or showing paracellular (through cell-cell junctions) or transcellular (inducing an invagination in a single HUVEC cell) TEM. We found that control Jurkat cells used transcellular and paracellular TEM routes; however, G9a depletion reduced significantly the number of cells undergoing both TEM types and increased the number of crawling cells (Figure 3c,d). Furthermore, by using BIX01294 we determined that blocking G9a for 1 h and 48 h also presented a significant increment in the number of crawling cells (Figure S3a,b). Together, these results indicate that G9a controls the ability of ALL to cross the endothelial barrier and extravasate into tissues. 

### 2.4. G9a Activity Does Not Affect Integrin Expression and Only Partially the Actin Polymerization in ALL Cells

**Figure 4:**
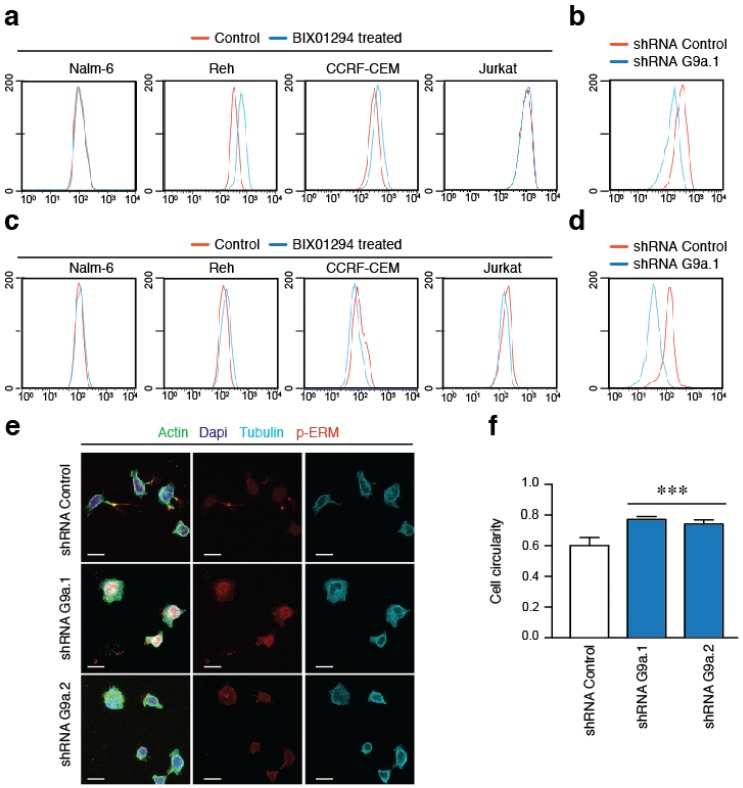
G9a does not affect integrin expression although might regulate cell morphology. (**a**) B- (Nalm-6 and Reh) and T- (Jurkat and CCRF-CEM) ALL cell lines were incubated with BIX01294 for 1h. Then, the expression of α4 subunits was determined by flow cytometry. Grey area represents untreated cells and green line BIX01294 treated cells; (**b**) The expression of the integrin subunit α4 was determined in control or G9a depleted Jurkat cells. Grey area represents control and green line G9a depleted cells; (**c**) B- and T-ALL cells lines were treated with BIX01294 for 1 h. Then, cells were fixed, permeabilized and their F-actin levels quantified by phalloidin staining; (**d**) Control and G9a depleted cells were processed as in (**c**) to quantified the levels of F-actin; (**e**) Control or G9a depleted Jurkat cells were cultured on VCAM1 (2 μg/mL) for 20 min. Cells were fixed and stained for tubulin (cyan) and trailing edge (phospho-ERM) marker; (**f**) Graph shows the rounded shape (circularity) of control or depleted cells for G9a in (**e**); Mean *n* = 3 replicates ± SD. Bar = 10 μm. *** *p* < 0.001.

We have previously shown that VLA-4 adhesion does not mediate G9a upregulation [17], however the effect of G9a inhibition on VLA-4 is currently unknown. We treated B- and T-ALL cell lines with BIX01294 for 1 h and then analyzed the expression of the integrin subunit α4. We detected variable expression levels of the integrin subunits without any down-regulation of these upon G9a inhibition compared with control cells (Figure 4a). Furthermore, by using a previously validated stable G9a depleted Jurkat cell line shRNA construct we only detected a slight reduction of the levels of α4 and β1 in G9a depleted Jurkat cells compared to control cells (Figure 4b). It has been described that G9a depletion does not affect cell adhesion [17]; however, we sought to examine the effect of G9a on F-actin polymerization. Remarkably, G9a inhibition did not diminished the levels of F-actin in any ALL cell lines (Figure 4c), whilst G9a depleted cells presented a reduction in the internal levels of polymerized actin (Figure 4d). Then, we analyzed the cellular morphology of G9a depleted cells cultured on VCAM-1. ERM (ezrin/radixin/moesin) proteins localizes at the rear pole of migrating lymphocytes [20]. Remarkably, control cells were able to polarize and present long trailing edge (stained for p-ERM) while G9a depletion promoted more rounded morphologies (Figure 4e,f). Moreover, G9a inhibition also affected the cellular morphology promoting more rounded forms (Figure S4a,b). Together, these results suggest that G9a targeting was not directly involved in integrin expression although it might be functionally relevant for ALL morphology and migration.

### 2.5. G9a Activity Modulates VLA-4-Mediated ALL Cell Migration

**Figure 5:**
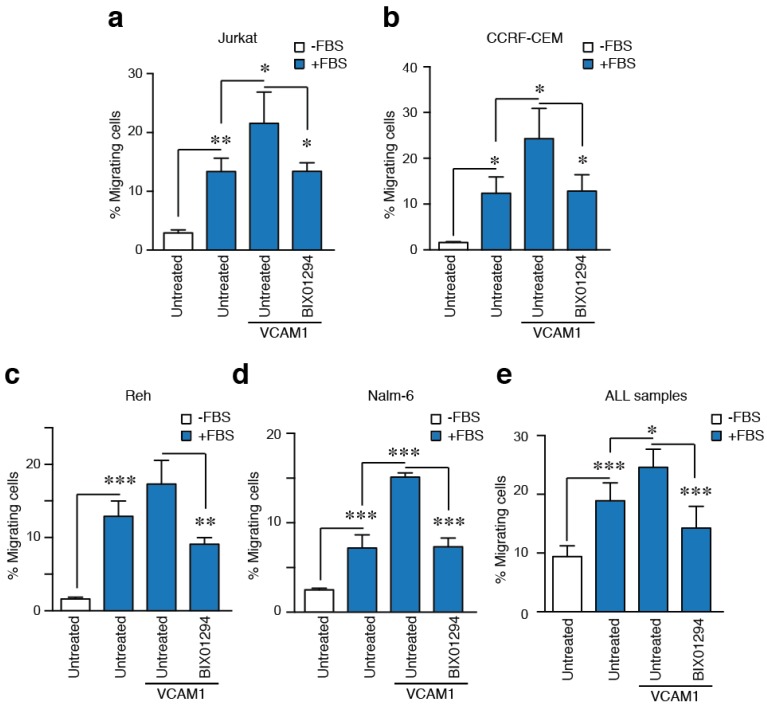
G9a inhibition impairs ALL cell squeezing induced by VLA-4 adhesion. (**a**) Jurkat; (**b**) CCRF-CEM; (**c**) Reh; (**d**) Nalm-6; and (**e**) primary samples from ALL patients were plated on upper chambers of Transwells of 3 μm of pore size. In some cases, the filter was previously coated with VCAM-1 (10 μg/mL). Serum was added at the bottom chamber to promote cell migration for 3 h. Then, cells were collected from the bottom chamber, stained and quantified. Mean *n* = 3 ± SD (for **a**–**d**) and Mean *n* = 5 ± SD (for **e**). * *p* < 0.05; ** *p* < 0.01; *** *p* < 0.001.

HUVEC cells express many molecules that contribute to TEM of normal and leukemic cells [21]. To discriminate how G9a is functionally involved in the VLA-4-mediated migration of ALL cells, we compared the ability of primary samples and ALL cell lines to cross through 3 μm pore transwells. All the ALL cell lines migrated robustly in response to serum, and this migration was enhanced when the transwell was previously coated with the ligand of VLA-4, VCAM-1. We confirmed the effect of G9a inhibition with BIX01294 in diminishing the migration induced by VLA-4 adhesion (Figure 5a–d). Likewise, when we studied migration of primary samples upon VLA-4 adhesion, we observed a significant increment in the migration index when cells attached to VCAM-1 (Figure 5e). Consistent with ALL cell lines, primary ALL cells treated with BIX01294 also reduced significantly the migration induced by VLA-4 adhesion (Figure 5e). Together, these results indicate that G9a activity is required during ALL cell movement.

## 3. Discussion

Cell trafficking into other tissues is critical during ALL dissemination, as the infiltration of leukemic cells into specific reservoirs, such as the CNS or testis, protects ALL cells from conventional therapies and promotes relapses in patients [22,23]. In this study, we have defined, for the first time the interplay between the integrin VLA-4 and the histone methyltransferase G9a in ALL cell migration. Overall, we aimed to gain more insight into the clinical relevance of G9a during the migration of leukemic cells of children with ALL. 

Current advances suggest a therapeutic role of epigenetic enzymes, such as DNA methyltransferases and histone deacetylases, with promising results for leukemia patients [6,24]. For instance, it has been suggested that the inhibition of the histone H3K27 demethylase UTX shows promising antitumor effects in T-ALL, while the activity of H3K27 methyltransferase EZH2 acts as tumor suppressor in ALL mouse models [25,26]. Other critical enzymes involved in tumor progression are the H3K9 methyltransferases. Suv39h1 activity controls cell migration in breast and colorectal carcinoma cells [27], while the depletion of suv39h2 induces apoptosis and cell death in ALL cells [28]. G9a is a histone methyltransferase overexpressed in multiple cancers and clear functions during lymphocyte development [11]. Recently, it has been published how G9a inhibition impairs cell cancer division and promotes cell death by inducing interferon-mediated genes and immunogenic response in several hematological malignancies, including AML and ALL [29]. In a complementary study, BIX01294 treatment upregulates p21 and pro-apoptotic members of Bcl-2 family [12]. Although these findings highlight the anti-tumor characteristics of G9a inhibitors, we now show in the present study that G9a inhibition might be relevant in ALL treatment not only by priming ALL for death, but also inhibiting the ability of leukemia cells to infiltrate other tissues.

The expression of VLA-4 is controversial in acute leukemias. In AML patients VLA-4 is a favorable marker associated with better prognosis [30]. In contrast, its expression is associated with poor prognosis in children, but not in adult, ALL patients [16,31]. Although the small number of healthy donors limits any conclusion about whether in normal lymphocytes G9a correlates or not with VLA-4, our results confirm a positive correlation between VLA-4 and G9a. In contrast to VLA-4, G9a expression presents an opposite trend with the clinical risk grade of patients, and when we stratified the patients according to their clinical risk, only the intermediate risk group presents a positive correlation between both molecules. In support of this, others have previously shown that basal levels of G9a or H3K9 methylation do not correlate with the tumor progression [29], although G9a targeting clearly might be relevant in the clinic. Our present results extend these previous findings, confirming that probably the functional connections between the integrin and G9a are more relevant in ALL than the expression of both molecules. H3K9 demethylases LSD1 and JMJD1A are related to tumor progression and metastasis in several cancer types, including colorectal and leukemias [32,33], as the balance between methylases and demethylases are critical for epigenetic changes, exploring these enzymes as potential therapeutic targets will have clinical interest in the future.

Our results also show that blocking the expression of G9a also resulted in a reduction of TEM in ALL cells. TEM of normal and leukemic cells involves several adhesive molecules, including selectins, ICAM-1, VCAM-1, PECAM-1 and others [21]. The functions of VLA-4 are central for the dissemination and drug resistance in ALL cells, and VLA-4 inhibition with blocking antibodies (Natalizumab) or small molecules (TBC3486) sensitizes leukemic cells to conventional therapies and improves survival in vivo models [34,35]. Recently, it has been shown that targeting VLA-4 with antisense drugs fails to improve survival in mouse model of ALL [36], supporting the idea that blocking the integrin and its molecular pathways has to continue being improved. Several molecules, such as myosin IIA, RhoA and Rap1b, translate integrins signals into cytoskeletal changes affecting the cell capacity to squeeze through HUVEC cells [37,38]. Also, other factors, such as MIP-β, contribute to the ability of the cells to cross the endothelial barrier [39]. Unlike these previous studies focusing on intracellular pathways, we show here that G9a, a nuclear component, could affect the nuclear morphology and migration of ALL cells onto a HUVEC monolayer.

The cell cytoskeleton and nuclear components are critical for nuclear deformability and cancer cell migration [40,41,42]. Moreover, integrins control the nuclear changes by translating external stimuli into the nucleus [43]. The effect of G9a inhibition on cell migration can be mediated by changes at gene transcription, nuclear deformability and intracellular signal levels. It has been previously demonstrated that G9a regulates several cell receptors, including integrins [44,45]. Although we have seen changes in cell morphology, they did not correspond to integrin expression changes or F-actin polymerization. The role of the nucleus during cell migration might be evaluated by forcing lymphocytes to move across pores sizes that require high nuclear deformability [42]. Our data clearly establish that G9a function was critical for ALL cell migration through narrow spaces across primary samples from patients and cell lines. We hypothesize that G9-mediated alterations of the chromatin structure may impair cell migration by affecting the physical properties of the nucleus and its connections with the cytoskeleton. The actin cytoskeleton is critical for cancer cell migration [46], and how its interactions with the nucleus regulate the nuclear deformability and transendothelial migration [47,48]. In agreement with this, it has been suggested that nuclear lobes and their cytoskeletal connections control the nuclear deformability of migrating neutrophils and lymphocytes [49]. Therefore, G9a induced mechanisms would be attractive for targeting leukemia dissemination and further pre-clinical evaluation of G9a inhibitors.

In summary, our results demonstrate novel cellular and functional connections between G9a and the ALL cell capacity to infiltrate in response to microenvironmental signals. Our observations indicate that targeting G9a clearly affects ALL cell migration, which might contribute to leukemia infiltration and dissemination through the patient’s body. In this context, studying the pathological relevance of G9a in ALL cell migration would be interesting to determine new therapeutic options in this leukemia.

## 4. Materials and Methods

### 4.1. Patients and Samples

A total of 50 ALL patients under 14 years old were retrospectively included in this study. ALL diagnosis and treatment was defined according to SEHOP-Pethema 2013 (Spanish Program for the Treatment of Hematologic Diseases). Primary human PBL were isolated from buffy coats of healthy anonymous donors (Blood Bank, Hospital Gregorio Marañón) after depletion of the monocyte fraction with CD14 microbeads. All guardians gave written informed consent. Patients were tested with conventional karyotyping and molecular studies using standard procedures. The total cohort of the study (*n* = 50) included seven relapses and six deaths. Patient characteristics are provided in Table 1.

### 4.2. Ethics Approval and Consent to Participate

Samples were obtained with informed consent for research purposes, and the procedures were approved by the Institutional Review Boards of the Hospital General Universitario Gregorio Marañón (Epicon) and the Hospital Universitario Niño Jesús (R0070/15). 

### 4.3. Cell Lines

The B- and T-ALL cell lines Jurkat, CCRF-CEM, Nalm6 and REH were obtained from Dr. Ramírez and cultured in RPMI 1640 with L-glutamine and 125 mM Hepes (Sigma Aldrich, St. Louis, MO, USA) with 10% fetal bovine serum (FBS, Sigma-Aldrich). HUVEC cells were obtained from Prof. Martin Humphries (The University of Manchester, Manchester, UK) and cultured in EBM-2 Endothelial Growth Basal Medium (Lonza, Walkersville, MD, USA). HUVECs were used up to the third passage. All cells were maintained in 5% CO_2_ at 37 °C.

### 4.4. Reagents and Antibodies

The mouse antibody anti-H3K9me2/3 (#5327), and the rabbit antibody -phospho-ERM (#3726) were from Cell Signaling (Danvers, MA, USA). The mouse antibody anti-β-tubulin (#T5201) was from Sigma-Aldrich. The antibody anti-α4 (HP2/1) was a gift from Prof. Sánchez-Madrid (Hospital de la Princesa, Universidad Autónoma de Madrid). The anti-ZO-1 antibody was from SantaCruz (Santa Cruz, CA, USA) (#sc-33725). Tetramethylrhodamine (TRITC)-Phalloidin, Alexa 647-Phalloidin, CellTrace™ CFSE, secondary antibodies Alexa-488, -594, -647 for immunofluorescence analysis and DAPI were obtained from Thermo Scientific (Waltham, MA, USA). VCAM1 was obtained from Peprotech. BIX01294 was from Abcam (Cambridge, UK).

### 4.5. RT- Real-Time PCR (qPCR)

Total RNA was extracted using the RNeasy kit (Qiagen, Valencia, CA, USA), and cDNA was synthesized from 1.0 μg of total RNA using SuperScript II First-Strand Synthesis System and oligo(dT) from Invitrogen (Thermo Scientific, Waltham, MA, USA). cDNA concentrations were quantified using a NanoDrop ND-1000 Spectrophotometer (Fisher Scientific, Pittsburgh, PA, USA). Oligonucleotides for selected genes were designed according to the Roche software (Universal Human Probe Roche Library, see supplementary material Table S1). Quantitative real-time PCR (qRT-PCR) was performed on a Roche LightCycler^®^ 480 following the manufacturer's instructions. Assays were made in triplicates and results normalized according to the expression levels of TBP (Roche Real Time Ready Single Assay ID 101145). Melt curve analysis was performed at the end of PCR to confirm the presence of a single, specific product. The results were expressed using the ΔΔ*C*_t_ method for quantification.

### 4.6. Production of Lentivirus

G9a was depleted with two different short hairpin RNA sequences. The previously reported shRNA G9a.1 [15] and G9a.2: (5′-GGACCTTCATCTGCGAGTATG-3′), which was purchased from Sigma-Aldrich and inserted in the pVenus lentiviral transfer vector (pVLVTHM). The recombinant lentiviruses were generated by transient transfection of 293T cells using Pei according to manufacturer’s protocol. Briefly, subconfluent HEK293T cells were co-transfected with the pVLVTHM vector and the packaging vectors pPsPax2, pMD2G. After 24 h, the medium was replaced with fresh medium 10 mM sodium butyrate and media changed after 6 h. 48 h later virus containing supernatants were harvested and filtered through 0.45 μm pore-sized membranes. Infection was performed by adding the lentiviral containing media to Jurkat cells at 1 × 10^6^ cells/mL, with 10 μg/mL Polybrene (Millipore, Zug, Switzerland). The media was changed after 24 h, and cells were passaged over two weeks. Stably infected cells were sorted by FACS based upon GFP fluorescence.

### 4.7. Immunofluorescence and Transendothelial Cell Migration

For stainings in 2D, control or G9a depleted Jurkat cells were cultured for 20 min on VCAM-1 (5 μg/mL). Cells were fixed in 4% formaldehyde (10 min), permeabilized with 0.5% Tx-100 in PBS (5 min), blocked in 10% fetal bovine serum and incubated with appropriated primary antibodies for 1 h at RT. After several washes, samples with incubated with secondary antibodies (1 h at RT). Samples were washed and mounted in Dako.

For transendothelial migration, HUVEC were plated on fibronectin-coated plates and confluent monolayers were stimulated with 15 ng/mL TNF-α, for 16 h prior to the assay. Then, HUVEC cells were labeled with CFSE and, after several washes, Control or G9a Jurkat cells were added to monolayer and allowed to transmigrate for 20 min. Cells were fixed, permeabilized and stained with appropriated antibodies. Images were acquired using a SP5 and SPE confocal microscopes (Leica, Wetzlar, Germany) with an ACS-APO 40× NA 1.30 oil immersion objective. We labeled control or G9a depleted cells with CFSE and cultured them on TNF-α stimulated HUVEC monolayer. Timelapes images were acquired with a TE2000 PFS microscope (Nikon, Tokyo, Japan) using a 20×/0.5 Plan Fluor objective and the Sedat filter set Chroma (89,000). The images were collected every 1 min over 30 min using a Cascade II EMCCD camera (Photometrics, Tucson, AZ, USA). Quantification of cell and nuclear circularity were determined using ImageJ (NIH, Bethesda, MA, USA). Track length was determined using Imaris (Bitplane, Zürich, Switzerland).

### 4.8. Flow Cytometry Analysis

Integrin VLA-4 expression was carried out by indirect immunofluorescence. Cells were blocked with human IgG (50 μg/mL; 30 min), incubated with HP2/1 (5–10 μg/mL; 30 min), followed by an appropriated fluorochrome-conjugated secondary antibody for 30 min (Jackson ImmunoResearch, West Grove, PA, USA). Between incubations, the preparations were washed with PBS. Flow cytometry was performed with a FacSort (Beckman Coulter, Brea, CA, USA) and data were analyzed using the BD CellQuest Pro software (BD Biosciences, Erembodegem, Belgium). To determine the content of polymerized actin (F-actin), cells were fixed with paraformaldehyde 4% in PBS for 15 min, permeabilized with Triton X-100 for 5 min and stained with (Alexa 647)-phalloidin (Molecular-Probes, Eugene, OR, USA). Cells were incubated at 22 °C for 10 min, washed twice with phosphate-buffered saline (PBS), and subjected to flow cytometry.

### 4.9. Transwell Invasion

We used transwell plate inserts (Corning Costar, 6.5 mm diameter, 3 μm pore sizes). In some cases, the filter was coated with 5 μg/mL of VCAM-1. 100 μL of a cell suspension (2 × 10^5^ cells/well) in serum free medium, preincubated or not with BIX01294, was added to the upper chamber. 600 μL of complete medium with serum was added to the bottom chamber to promote the cell migration. The chambers were incubated in a CO_2_ incubator at 37 °C for 6 h. Migrated cells from the lower chamber were collected, stained and quantified. 

### 4.10. Statistical Analysis

Student *t* test (two tailed Mann-Whitney non-parametric test) or ANOVA (two tailed Kruskal-Wallis non-parametric test) were used for between-group analysis. For all analyses, statistical calculations were performed using Prism 6.0 Software (GraphPad Software, Inc. La Jolla, CA, USA), and *p*-values < 0.05 were considered statistically significant.

## 5. Conclusions

Here, we provide the first evidence for the functional involvement of G9a activity in the migration of primary ALL cells from patients. Although we observed a correlation between the integrin VLA-4 and the expression of G9a, the number of patients and healthy donors is not sufficient to conclude the potential interest of G9a as biomarker in this pathology and further studies might be conducted in the future. This study provide direct evidence that G9a depletion or inhibition disrupts cell polarity and cell capacity to extravasate endothelial barriers or squeeze through narrow spaces. Taken together, our findings indicate that G9a contributes to leukemia cell migration and might be considered a potential therapeutic target to block cancer cell dissemination.

## Figures and Tables

**Table 1:** Clinical and biological characteristics of the studied ALL patients.

Variable	Characteristics	Number of Cases	Percentage (%)
Gender	Male	27	54
	Female	23	46
Risk group	1 (LR ^1^)	11	22
	2 (IR ^1^)	32	64
	3 (HR ^1^)	7	14
WBC ^1^ (>50,000 cells/mm^3^)	Positive	6	12
	Negative	44	88
MRD ^1^ after induction	Positive	22	44
	Negative	28	56
Extramedullary disease	Positive	22	44
	Negative	28	56
Blast in BM ^1^	Positive	25	50
	Negative	25	50
ETV6/RUNX1	Positive	15	30
	Negative	32	64
	ND ^1^	3	6
BCR/ABL	Positive	2	4
	Negative	47	94
	ND ^1^	1	2
MLL rearrangements	Positive	2	4
	Negative	47	94
	ND ^1^	1	2
Hyperdiploidy	Positive	13	26
	Negative	36	72
	ND ^1^	1	2
Relapse	Positive	7	14
	Negative	41	82
	ND ^1^	2	4
Death	Positive	6	12
	Negative	43	86
	ND ^1^	1	2

^1^ Abbreviations: ALL, acute lymphoblastic leukemia; BCR, breakpoint cluster region; ABL, Abelson; MLL, mixed-lineage leukemia; LR, low risk; IR, intermediate risk; HR, high risk; WBC, white blood cells count at diagnosis; MRD, minimal residual disease after induction; BM, bone marrow; ND Not determined.

**Table 2:** G9a expression according to risk group.

Risk Group	G9a LE ^1^	G9a HE ^1^	Percentage (%)
1	3	8	27.3/72.7
2	17	15	53.1/46.9
3	5	2	71.4/28.6

^1^ Abbreviations: LE, low expression (lower than the median); HE, high expression (higher than the median).

## Supplementary Materials

The following are available online at http://www.mdpi.com/2072-6694/10/9/325/s1. The supplementary material enclosed to this manuscript includes: one Supplementary Table S1, five Supplementary Videos, and four Supplementary Figures. Figure S1: Correlation between SUV39H1 and ITGA4 in ALL patients and between G9a and ITGA4 in healthy donors. Figure S2: Cell attachment and nuclear spreading of Jurkat cells treated with BIX01294 on a monolayer of HUVEC cells. Figure S3. Transendothelial migration of Jurkat cells treated with BIX01294. Figure S4. Cell polarity of Jurkat cells treated with BIX01294. Table S1. Oligonucleotides for selected genes.
