# Supplementary material for: G9a Correlates with VLA-4 Integrin and Influences the Migration of Childhood Acute Lymphoblastic Leukemia Cells
Source: Cancers (Basel). 2018 Sep 12;10(9):325. doi: 10.3390/cancers10090325 (PMC6162492; doi:10.3390/cancers10090325)
Supplement: Supplementary file 1 [file cancers-10-00325-s001.zip › cancers-345053-supplementary/cancers-345053-supplementary.pdf]

cancers

MDPI

Supplementary Materials

G9a correlates with VLA-4 integrin and influences the migration of childhood acute lymphoblastic leukemia cells.

Elena Madrazo, David Ruano, Lorea Abad, Estefanía Alonso-Gómez, Carmen Sánchez-Valdepeñas, África González-Murillo, Manuel Ramírez, and Javier Redondo Muñoz.

Table S1. Oligonucleotides for selected genes.

Gene name	Sense	Sequence
ITGA4	Forward	GCGTGGTACAACTTGACTGC
	Reverse	TCCTCTTCCGCTCTGCTG
G9A	Forward	GGACACCCCTCGTAGTGAAG
	Reverse	GACAGAGGCTGGAGATGAGG
SUV39H1	Forward	GTCATGGAGTACGTGGGAGAG
	Reverse	CCTGACGGTCGTAGATCTGG

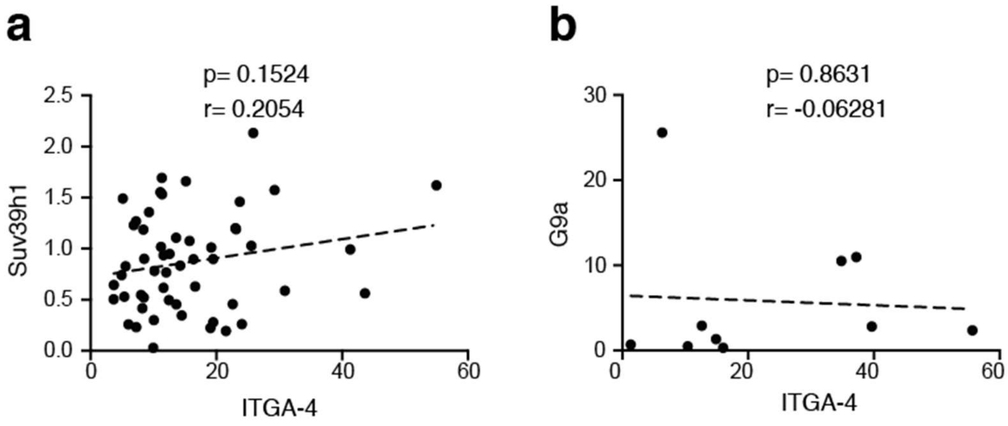

Figure S1 (**a**) ITGA-4 and Suv39h1 expression analyzed by RT-qPCR. Expression levels were normalized by TBP and graph shows the mean of children ALL patients (n = 50). Pearson's correlation coefficient (*r*) and *p*-value are shown. (**b**) Samples from healthy donors (n = 10) were analyzed for the correlation between ITGA-4 and G9a. Pearson's correlation coefficient (*r*) and *p*-value are shown.

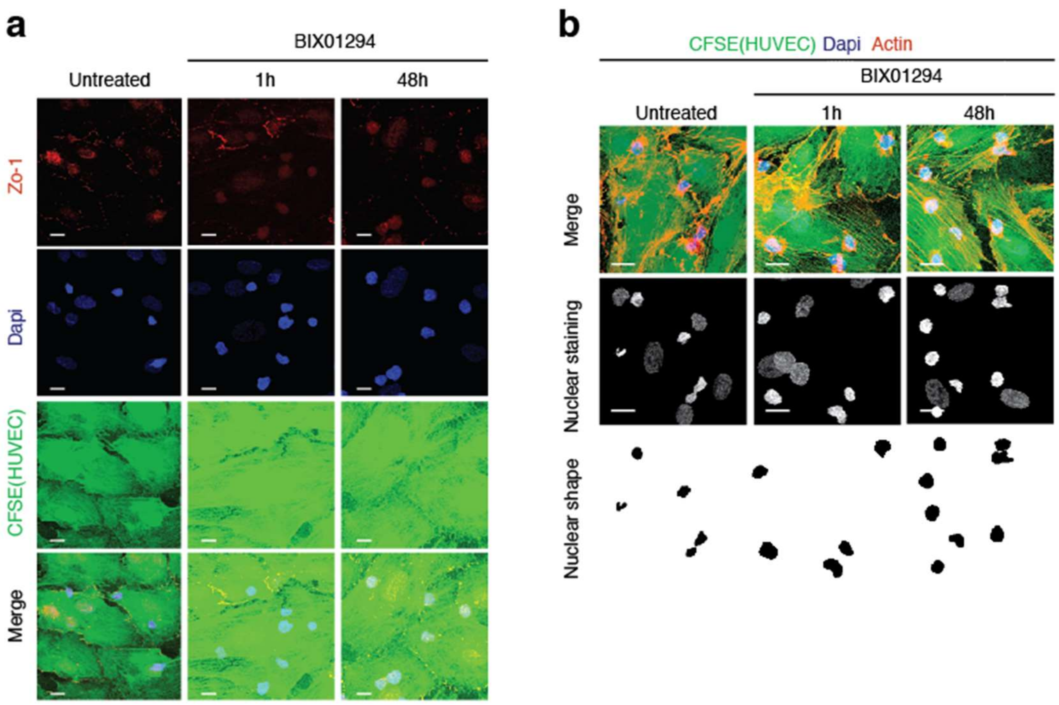

Figure S2 (**a**) HUVEC cells were grown to confluency, labelled with CFSE and stimulated with TNFα for 16h. Control or BIX01294-treated Jurkat cells were plated on TNFα-activated HUVEC cells. Cells were fixed, permeabilized and analyzed to visualized their nuclei (DAPI, blue), F-actin (Phalloidin, cyan), and endothelial junctions (Zo-1, red). Bar= 10 µm. (**b**) Control and BIX01294-treated Jurkat cells were cultured on CFSE labelled HUVEC activated with TNFα, fixed and stained for DAPI (blue) and F-actin (red). Nuclear shapes were determined. Bar= 10 µm.

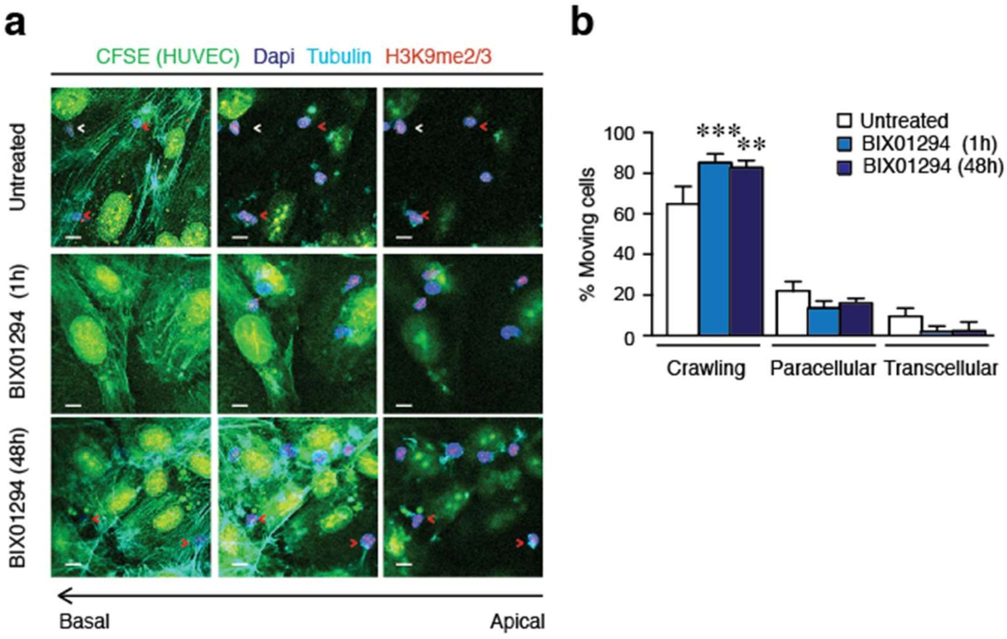

Supplementary Figure S3. (**a**) Control or BIX01294-treated Jurkat cells were plated on TNFα-activated HUVEC cells labelled with CFSE. After 30 min, cells were fixed, permeabilized and analyzed to visualize their nuclei (blue), F-actin (cyan), and H3K9me2/3 (red). White arrows indicate cells undergoing transcellular TEM. Red arrows indicate cells crossing through cell-cell junctions in paracellular TEM. (**b**) Graph shows the percentage of control or BIX01294-treated cells crawling or performing TEM. Mean n=3 ± SD. Bar= 10 µm. $p<0.01$ **; $p<0.001$ ***.

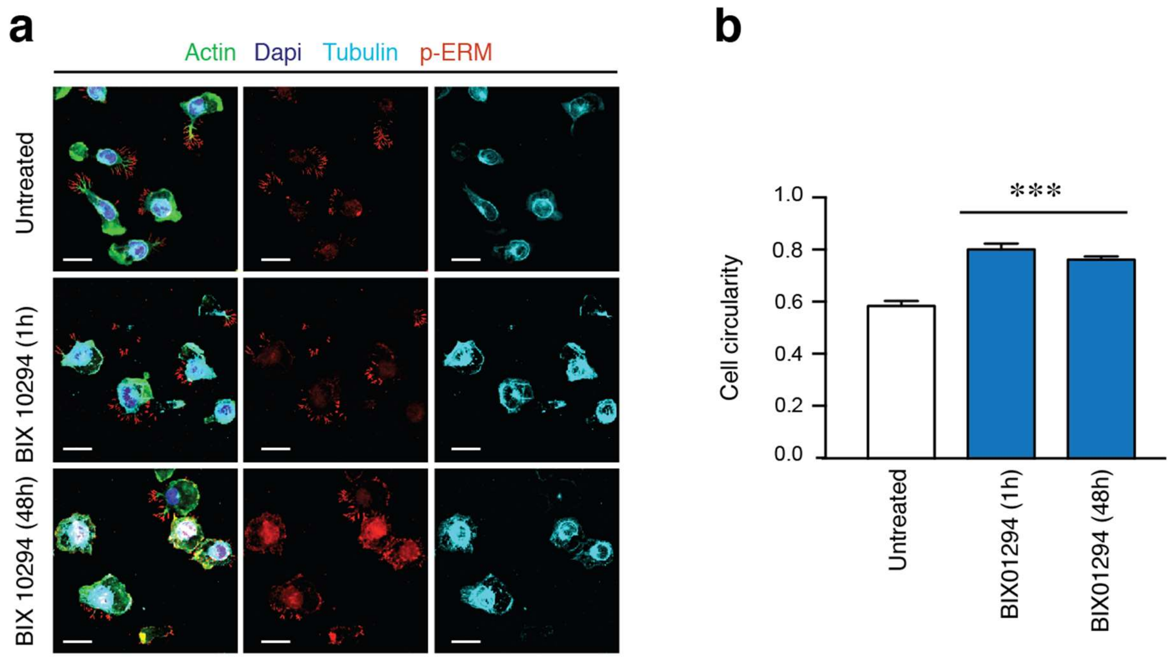

Supplementary Figure S4. (**a**) Control or BIX01294-treated Jurkat cells were cultured on VCAM1 (2 µg/ml) for 20 min. Cells were fixed and stained for tubulin (cyan) and trailing edge (phospho-ERM) marker. (**b**) Graph shows the rounded shape (circularity) of control or BIX01294-treated cells. Mean n=3 replicates ± SD. Bar= 10 µm. p<0.001 ***.
